# Association between health worker motivation and healthcare quality efforts in Ghana

**DOI:** 10.1186/1478-4491-11-37

**Published:** 2013-08-14

**Authors:** Robert Kaba Alhassan, Nicole Spieker, Paul van Ostenberg, Alice Ogink, Edward Nketiah-Amponsah, Tobias F Rinke de Wit

**Affiliations:** 1 Noguchi Memorial Institute for Medical Research, University of Ghana, Legon, Ghana; 2 Amsterdam Institute for Global Health and Development, University of Amsterdam, Amsterdam, Netherlands; 3 PharmAccess Foundation, Amsterdam, Netherlands; 4 Joint Commission International (JCI), Chicago, USA; 5 Department of Economics, University of Ghana, Legon, Ghana

**Keywords:** Ghana, Health worker, Motivation, Quality care, Primary health facilities, Patient safety, Efforts

## Abstract

**Background:**

Ghana is one of the sub-Saharan African countries making significant progress towards universal access to quality healthcare. However, it remains a challenge to attain the 2015 targets for the health related Millennium Development Goals (MDGs) partly due to health sector human resource challenges including low staff motivation.

**Purpose:**

This paper addresses indicators of health worker motivation and assesses associations with quality care and patient safety in Ghana. The aim is to identify interventions at the health worker level that contribute to quality improvement in healthcare facilities.

**Methods:**

The study is a baseline survey of health workers (n = 324) in 64 primary healthcare facilities in two regions in Ghana. Data collection involved quality care assessment using the SafeCare Essentials tool, the National Health Insurance Authority (NHIA) accreditation data and structured staff interviews on workplace motivating factors. The Spearman correlation test was conducted to test the hypothesis that the level of health worker motivation is associated with level of effort by primary healthcare facilities to improve quality care and patient safety.

**Results:**

The quality care situation in health facilities was generally low, as determined by the SafeCare Essentials tool and NHIA data. The majority of facilities assessed did not have documented evidence of processes for continuous quality improvement and patient safety. Overall, staff motivation appeared low although workers in private facilities perceived better working conditions than workers in public facilities (*P* <0.05). Significant positive associations were found between staff satisfaction levels with working conditions and the clinic’s effort towards quality improvement and patient safety (*P* <0.05).

**Conclusion:**

As part of efforts towards attainment of the health related MDGs in Ghana, more comprehensive staff motivation interventions should be integrated into quality improvement strategies especially in government-owned healthcare facilities where working conditions are perceived to be the worst.

## Background

Universal access to good quality care and optimal patient safety is the goal of health systems and governments all over the world. Even though developed countries have made significant achievements towards attainment of this goal, many developing countries in Africa lag behind due to financial, material and human resource constraints.

Of the estimated global health workforce of 59.2 million, 3% are found in Africa coping with 25% of the global disease burden. It is estimated that the health sector workforce density per 1000 population in Africa is 2.3 compared to 24.8 in the Americas [1].

Ghana is one of the sub-Saharan African countries making considerable progress in many health outcome indicators. For instance, the percentage of antenatal and postnatal coverage improved from 42.2% and 33.8% in 2008 to 91.3% and 64.7% in 2011, respectively [2]. The percentage of deliveries attended by skilled health staff also increased from 44.2% in 2008 to 52.3% in 2011. Likewise, the number of outpatient visits per capita improved from 0.77 in 2008 to 1.07 in 2011.

However, these achievements are insufficient to attain the 2015 targets for the health related Millennium Development Goals (MDGs) [3]. This is due to a number of factors, including understaffing in health facilities, inequitable distribution of health sector human resources, de-motivated staff and inadequate healthcare infrastructure [3,4]. To attain these health related MDGs, there is the need for more comprehensive quality improvement interventions including a health sector human resource (HSHR) approach.

An estimated 52 258 people are formally working in the health sector in Ghana, of which 81.5% are employed in the public sector serving more than 24 million people [5]. Of the formal sector workers, 56% are non-clinical staff while 44% are clinical staff. There are also 21 791 people registered as engaged in traditional medicine and 367 registered traditional birth attendants (TBAs) [5].

Even though doctor-patient and nurse-patient ratios have progressively improved over the years, rural–urban inequities still exist, with substantially more unfavorable ratios in the rural areas. The doctor-patient ratio in 2006 was 1:14 733 but improved to 1:10 034 in 2011. Likewise, nurse-patient ratio improved from 1:1537 in 2006 to 1:1240 in 2011 [2]. Two major teaching hospitals in urban Ghana employ more than 45% of the country’s medical doctors. Fewer than 15% of medical doctors work in the district and sub-district health facilities. In total, an estimated 68% of the health workforce works in urban areas and 32% in the rural areas where more than 50% of the Ghanaian population lives [5].

Governance and professional regulation of health sector human resources in Ghana is within the domain of the Human Resources for Health Development Directorate (HRHDD) under the Ministry of Health (MOH). Professional bodies such as the medical and dental council, nurses and midwives council, and the pharmacy council are government institutions also mandated to ensure professional competence and discipline of members during and after training.

Among the key functions of the HRHDD are: policy strategy; planning and distribution of health staff; coordination of pre-service training with relevant training institutions; development of staff training functions; and monitoring and evaluation.

The setting of this study was in the Greater Accra (GAR) and Western (WR) regions of Ghana. The GAR is predominantly urbanized and cosmopolitan with close to 4 million people and 288 National Health Insurance Authority (NHIA) accredited healthcare facilities. The region has an estimated 4209 nurses, 1186 midwives and 1107 doctors. This represents 31%, 33% and 64% of Ghana’s total population of nurses, midwives and doctors, respectively [5]. The GAR is the national capital of Ghana and has 10 administrative districts.

The WR is predominantly rural with a population of a little over 2 million people and 292 NHIA accredited healthcare facilities. The region has an estimated 1484 nurses, 380 midwives and 124 doctors, representing 10.8%, 10.6% and 7.1% of the country’s total population of nurses, midwives and doctors, respectively [5]. The WR has 17 administrative districts.

This study is necessitated by the relatively minimal attention given to boosting staff motivation as a quality improvement strategy. Until recently, health worker motivation as a healthcare quality improvement strategy was not emphasized in health sector reforms in most countries [6,7]. Nonetheless, HSHR is an important input in quality healthcare delivery and the pillar of every health system [1,8,9].

In addition, low staff motivation can be a major contributing factor to poor service quality in healthcare facilities and will likely be associated with staff impatience to clients, absenteeism, long waiting times, informal fee charges and increased labour strike actions [1,4,10,11].

The current paper is aimed at exploring the quality care and patient safety situation in health facilities accredited by the Ghanaian NHIA (government regulatory body) and identify associations with staff motivation. This would contribute to identifying priority areas of interventions at the health worker level to improve safety and quality care for clients and contribute to increased willingness to (re-)enroll in the national health insurance scheme (NHIS).

According to Ghana’s NHIS law (Act 650), health care facilities willing to render services to NHIS insured clients must be accredited by the NHIA for five years on the first account, subsequently renewable every two years. Depending on facility performance during on-site assessment and inspection, an accreditation outcome can be full, selective, provisional or denied. Full accreditation is granted when a facility attains a total mean percentage assessment score of at least 50% which is equivalent to grade D.

## Methods

### Study design and data collection

This study comprises a baseline longitudinal survey of staff motivation aspects as well as staff efforts in patient risk reduction and quality care improvement using semi-quantitative data collection techniques. Structured questionnaires were developed on staff workplace motivational factors based on preceding in-depth interviews with health workers.

Respondents were asked to rank their level of satisfaction with 19 workplace motivating factors on a four-point Likert scale from 1 = “very disappointing” to 4 = “very satisfactory”. Additional questions were asked on workers’ socio-demographic characteristics, financial responsibilities, financial status and additional sources of income besides regular employment income.

Apart from the structured questionnaire for staff, the NHIA accreditation data^a^ and the ESS patient risk assessment data^b^ were used to ascertain the quality care situation in the sampled facilities.

This study supplemented the NHIA data with the SafeCare Essentials (ESS) patient risk assessment tool because the former does not emphasize staff behaviour, attitudes and efforts toward quality care and patient risk reduction which is the main focus of this study. Moreover, at the time of conducting this study the researchers did not have access to up-to-date disaggregated NHIA accreditation scores on the sampled 64 facilities. In view of this, aggregate NHIS scores in five core areas were analysed alongside scores of the ESS tool.

To control for bias during administration of the ESS tool, scoring was done by three trained research assistants who agreed on final scores for every facility after independent scoring. As part of the assessment process, clinic administrative records were reviewed alongside observations and key informants’ interviews.

The semi-structured staff questionnaires and ESS tool were piloted in two conveniently sampled clinics in the GAR. The piloting allowed for correction of typographical mistakes and conversance with the interview process.

### Sampling procedures

The GAR and WR were purposively sampled for rural–urban balance and to avoid spillover effects during implementation of interventions, since both regions do not share a common boundary. In each region, eight districts were randomly sampled using Principal Component Analysis (PCA) [12]. At the health facility level, PCA was used to generate scores for NHIS accredited primary healthcare facilities in the GAR and WR. Using the quota sampling system, each selected district in a region was allocated a maximum of four qualified health facilities. Per this criterion, 32 public and private facilities were randomly sampled from each region to make a total of 64.

The sampled 64 facilities represent a little more than 2% of the total number of accredited facilities (n = 2647) in Ghana as of 2010. The 32 facilities from each region represent 11% of the total number of accredited facilities in GAR (n = 288) and WR (n = 292).

At the staff level, clinical and non-clinical health workers were interviewed from all 64 facilities. Inclusion criteria were full time employment and at least six months work experience to elicit responses from staff who are more experienced and better informed about their work environment. To avoid professional skewing in responses and ensure reliability, at most one respondent from each professional category was randomly sampled and interviewed in the selected health facilities.

### Ethical considerations

Ethical clearance for the surveys was obtained from the Ghana Health Service (GHS) Ethical Review Board (ERB) (clearance number: GHS-ERC: 18/5/11). Informed consent was obtained from health facility heads, the district and regional health directorates and individual respondents.

### Statistical analysis

All data sets were analysed with Stata statistical software (version 12.0) after data cleaning and coding to anonymize facilities and staff. To ensure internal validity, all questions were informed by research objectives and reviewed literature. Cronbach’s alpha (α) was conducted to check for scale reliability of the 19 Likert scale items on workplace motivating factors and found to be 0.82, which is above the 0.70 rule of thumb [13,14]. Moreover, different cadres of health professionals were interviewed to ensure external validity of the findings.

Parametric and non-parametric tests were used in the data analysis and hypothesis testing. Factor analysis was first conducted with orthogonal varimax rotation (Kaiser off) to group the 19 workplace motivational factors into 4 major factors [12].

Based on Bennette and Franco’s [7] conceptual framework, these four factors were predicted and named as follows: (1) clinic physical work environment (clinic physical environment, attitude of superiors, attitude of colleague workers and workload in clinic); (2) resource and drugs availability (availability of adequate modern equipment, availability of consumables/logistics, number of clinic staff, water supply, electricity supply and availability of drugs for patients); (3) financial and extrinsic incentives (monthly salary, payment of financial incentives, reputation and recognition from job, accommodation for staff, transportation, allowance for staff, client responsiveness to staff instructions and shuttle transport for staff); and (4) job prospects and career development (possibility for promotion and opportunity for further education).

Following the factor analysis, Spearman’s ranked order correlation test was conducted to ascertain the association between the clinic’s level of effort towards quality care and staff motivation levels in the four factor-analysed staff motivational markers. This statistical analysis also tested the hypothesis that level of health worker motivation is associated with level of effort by primary healthcare facilities to improve quality care and patient safety.

## Results

### Characteristics of health workers

A total of 333 questionnaires were administered to health workers in 64 clinics, of which 324 were correctly completed and returned, representing a 97% return rate. Of the workers interviewed, 56% were from rural facilities, 44% were from urban facilities, 57% worked in private facilities and 43% worked in public facilities. The majority of the respondents were female (67%) and the mean age was 39 years (SD = 14, 95% CI = 37–40). Most of the workers had tertiary (46%) and secondary education (34%). The majority of respondents (272; 84%) were clinical staff, mostly not married (57%) and Christians (96%).

Staff 40-years-old or younger were within the lower monthly salary bracket than other age groups (*P* <0.001). Likewise, workers with at least tertiary education received higher salaries than those with secondary education (*P* <0.001). Clinical staff and unmarried staff were in the higher income bracket than non-clinical and married workers (*P* <0.001) (See Table 1).

**Table 1:** Characteristics of health workers (n = 324)

	**Facility ownership**			**Geographical location**			**Range of monthly salary**			**Total**
	**Private**	**Public**	***P*-value**	**Rural**	**Urban**	**P-value**	**<GHC 500**	**>GHC 500**	***P*-value**	
**Variables**	**f (%)**	**f (%)**		**f (%)**	**f (%)**		**f (%)**	**f (%)**		**f (%)**
Gender										
Male	66(20)	41(13)	0.242	64(20)	43(13)	0.354	52(16)	55(17)	0.077	**107(33)**
Female	119(37)	98(30)		118(36)	99(31)		89(27)	128(40)		**217(67)**
Age										
**≤**40 years	111(34)	80(25)	0.658	118(36)	73(23)	**0.015**^**a**^	135(42)	56(17)	**0.000**^**a**^	**191(59)**
41-60 years	44(14)	49(15)	**0.024**^**a**^	44(14)	49(15)	**0.041**^**a**^	31(10)	62(19)	**0.000**^**a**^	**93(29)**
**≥**61 years	30(9)	10(3)	**0.015**^**a**^	20(6)	20(6)	0.401	14(4)	26(8)	**0.005**^**a**^	**40(12)**
Education										
Secondary	66(20)	46(14)	0.629	67(20)	45(14)	0.336	64(20)	48(14)	**0.001**^**a**^	**112(34)**
Tertiary	119(37)	93(29)		115(36)	97(30)		80(25)	132(41)		**212(66)**
Profession category										
Clinical^b^	154(48)	118(36)	0.689	151(47)	121(37)	0.585	135(42)	137(42)	**0.000**^**a**^	**272(84)**
Non-clinical^c^	31(10)	21(6)		31(10)	21(6)		9(3)	43(13)		**52(16)**
Marital status										
Married	79(24)	61(19)	0.832	67(21)	73(22)	**0.009**^**a**^	78(24)	62(19)	**0.000**^**a**^	**140(43)**
Not married	106(33)	78(24)		115(36)	69(21)		66(20)	118(37)		**184(57)**
Religion										
Christian	179(55)	133(41)	0.613	176(54)	136(42)	0.661	175(54)	137(42)	0.324	**312(96)**
Other	6(2)	6(2)		6(2)	6(2)		5(2)	7(2)		**12(4)**

^a^Statistically significant at 0.05 level of significance. Source: COHEISION Project Clinic Staff Interviews Data (March-June, 2012). GHC, Ghana cedi.

^b^Clinical staff includes medical staff who have direct contact with patients. They include medical doctors, nursing personnel, pharmacy personnel, medical assistants and laboratory personnel.

^c^Non-clinical staff are the support staff who do not have direct contact with patients. They include administrators, accountants, labourers and receptionists.

### Staff experiences and level of motivation by work conditions

To ascertain the conditions under which health workers perform their duties and how these conditions either constrain or motivate them to deliver good quality care, respondents were asked questions related to their mode of transport to work, working hours, workload and monthly salaries.

The dominant mode of transport to work on a daily basis was walking (46%) followed by public transport (38%) and use of personal car (12%). More than half (52%) of the respondents indicated that they regularly report to work late. Of this number, 78% of them do so once a week; 22% report to work late twice or more times a week.

Results on the financial status and responsibilities of health workers showed that 55% of the staff interviewed earned less than GHC 500 as a monthly salary; 40% earned between GHC 500 and GHC 1300; 4% earned more than GHC 1300. Only 16% of the respondents indicated they receive an additional monthly work allowance from their clinics. Apart from their regular employment, 30 respondents (9%) said they were engaged in additional income generating work (moonlighting).

As shown in Table 2, most of the respondents (58%) said they were members of at least one professional association, with a minority (5%) playing active roles as chairperson, patron or treasurer. The majority of the health workers (58%) who belonged to at least one professional association indicated that membership in these associations positively influenced their professional practice (See Table 2).

**Table 2:** Staff experiences with work conditions, transportation to work and affiliation with professional associations

	**Statistics**		
**Variables**	**Obs.**	**Frequency (f)**	**Percentage (%)**
Regular mode of transport to work	324		
Walk		149	46
Bicycle		4	1
Motor cycle		5	2
Public transport		124	38
Personal car		38	12
Missing system		4	1
Occasional tardiness to work	320		
Yes		165	52
No		155	48
Number of times staff reports to work late a week	164		
Once		128	78
Twice or more		36	22
Range of monthly salary^a^ received from clinic	324		
<GHC 500		180	55
GHC 500- GHC 1300		128	40
>GHC 1300		13	4
Missing system		3	1
Work allowance received from clinic outside monthly salary	311		
Yes		51	16
No		260	84
Additional work(s) outside permanent work in clinic	312		
Yes		30	9
No		282	87
Missing system		12	4
Membership in formal professional association	241		
Yes		140	58
No		101	42
Role in professional association	133		
Just a member		127	95
Chairperson		1	1
Patron		1	1
Treasurer		4	3
Membership in professional association influence professional practice	191		
Yes		110	58
No		81	42

Source: COHEISION Project Clinic Staff Interviews Data (March-June, 2012). Obs., Number of observations.

^a^US$ 1.0 is equivalent to 2.1 Ghana *Cedis* (GHC): (XE.com/currency converter, 13/08/2013).

An independent t-test was conducted to test for differences in mean scores on rated satisfaction with the factor-analysed working conditions. It was found that workers in private health facilities were more motivated by the physical work environment of their clinics (mean score = 3.2) than those in public facilities (mean = 2.3), *P* <0.001. In addition, health workers in private facilities expressed better satisfaction with availability of drugs and other medical resources (mean = 3.1) than those in public facilities (mean = 2.8), *P* <0.05.

Even though overall satisfaction with financial and extrinsic incentives was low in all the 64 facilities, staff in public health facilities expressed greater dissatisfaction (mean = 1.9) than their counterparts in private facilities (mean = 2.2). In addition, workers in urban facilities said they were more satisfied with drug and resource availability in their facilities than workers in rural facilities (*P* <0.05) (See Table 3).

**Table 3:** Differences in staff satisfaction with four aggregate markers of staff motivation

**Aggregated motivating factors**	**Facility ownership**			**Geographic location**		
	**Private**	**Public**	***P*-value**	**Rural**	**Urban**	***P*-value**
	**Mean(SD**^**b**^**)**	**Mean(SD)**		**Mean(SD)**	**Mean(SD)**	
Physical work environment (n = 318)	3.2(0.47)	2.3(0.51)	0.0000^**a**^	3.0(0.51)	3.1(0.48)	0.1630
Availability of resources and drugs (n = 321)	3.1(0.75)	2.8(0.77)	0.0000^**a**^	2.8(0.81)	3.1(0.75)	0.0019^**a**^
Financial and extrinsic^c^ incentives (n = 312)	2.2(0.64)	1.9(0.55)	0.0002^**a**^	2.0(0.63)	2.1(0.60)	0.8302
Job prospects and career development (n = 308)	2.4(0.86)	2.5(0.80)	0.1539	2.5(0.82)	2.3(0.86)	0.1772

^a^Statistically significant at 0.05 level of significance. Source: COHEISION Project Clinic Staff Interviews Data (March-June, 2012).

^b+^SD = Standard deviation. Mean scores and SD are based on a 4 point Likert scale from 1 = very disappointing to 4 = very satisfactory. Higher mean scores SD, therefore, suggest higher staff satisfaction with workplace motivating factors and vice versa.

^c^Extrinsic incentives include prestige and societal recognition gotten from the job.

### Quality care situation in sampled health facilities

**Figure 1:**
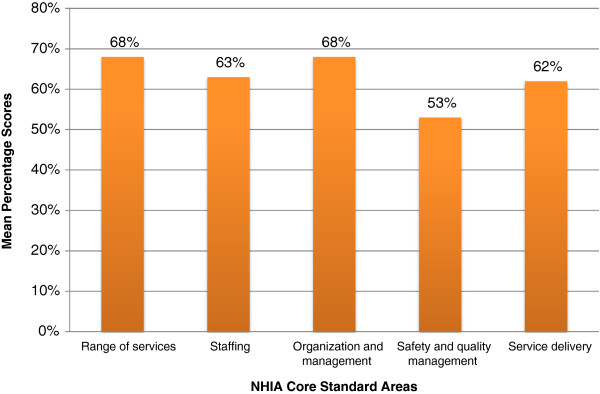
^**++**^**Mean percentage scores in five NHIA core standard areas (n = 64). **^++^Mean percentage scores: calculated by adding all applicable criteria (0 – 3) scores under each standard area divided by the total expected score per standard area and multiplied by 100. Source: NHIA Accreditation data for 64 sampled health facilities (2009–2011). Legend: NHIA (National Health Insurance Authority).

Analysis of the NHIA accreditation data showed that among the five core standard areas, safety and quality management was the area where most facilities performed worst (mean percentage score = 53%). Performance was however better in: range of services, organization and management, and staffing and service delivery. Details of the mean percentage scores of the 64 facilities in the five core standard areas are shown in Figure 1.

**Figure 2:**
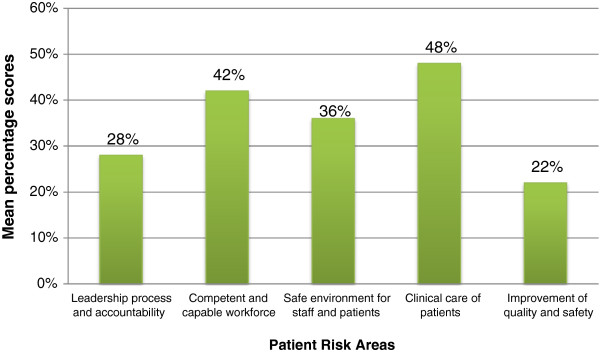
^**+++**^**Mean percentage scores in essentials’ five patient risk areas (n = 64).** +++Mean percentage scores: calculated by adding all applicable criteria (0 – 3) scores under each risk area divided by the total expected score per risk area and multiplied by 100. Source: COHEISION Project Patient Risk Assessment Using the Essentials Tool (March-June, 2012).

Apart from the NHIA accreditation data on the quality situation of the surveyed facilities, the Essentials patient risk assessment tool was used to ascertain the levels of effort of these health facilities towards risk reduction and safety. Results of the assessment showed that overall performance of the facilities was low with none of therisk areas attaining a mean percentage score up to 50%. Quality improvement and patient safety was the area with the lowest score (mean percentage score = 22%) followed by leadership processes and accountability (mean percentage = 28%). Clinical care of patients and competency of workforce were areas with better mean percentage scores of 48% and 42% respectively (See Figure 2).

### Association between staff motivation and quality care in health facilities

Spearman’s rank order correlation test was conducted using the four aggregate markers of staff motivation and quality care standards according to the ESS and NHIA tools to ascertain the association between staff motivation levels and quality care in health facilities.

The results showed a positive correlation between staff motivation levels and clinics’ efforts towards quality improvement and patient safety (See Table 4). Most of the quality care indicators according to the ESS tool positively correlated with the four aggregate staff motivation factors (*P* <0.05). The NHIA quality care standard areas were positively correlated with staff satisfaction level with financial incentives and job prospects (*P* <0.05) (See Table 4).

**Table 4:** Association between staff motivation and quality healthcare

	**Staff motivation factors**^**b**^			
**ESS and NHIA quality care markers**	**Motivating factor 1**	**Motivating factor 2**	**Motivating factor 3**	**Motivating factor 4**
**ESS patient risk areas**	**Coef.**	**Coef.**	**Coef.**	**Coef.**
Leadership and accountability	0.2702^a^	0.3013^a^	0.2538^a^	0.0896
Competency of workforce	0.1616^a^	0.1258^a^	0.2411^a^	0.1788^a^
Environmental safety	0.1086	0.1306^a^	0.1600^a^	0.0452
Clinical care	0.2621^a^	0.2273^a^	0.2918^a^	0.1526^a^
Quality improvement	0.1776^a^	0.2387^a^	0.2952^a^	0.2463^a^
**Overall quality care**	0.2478^**a**^	0.2716^**a**^	0.2764^**a**^	0.1110^**a**^
**NHIA core standard areas**	**Coef.**	**Coef.**	**Coef.**	**Coef.**
Range of services	−0.1851^a^	−0.1731^a^	−0.1352^a^	−0.0129
Staffing	−0.0847^a^	−0.1219^a^	−0.0099	0.0544
Organization and management	0.0289	−0.1357^a^	0.1057	0.0858
Quality and safety management	0.0419	0.0207	0.1475^a^	0.1250^a^
Care delivery	−0.0821	−0.0299	0.0854	0.0601
**Overall quality care**	−0.0258	0.0271	0.0760	0.1236^**a**^

^a^Spearman correlation coefficient statistically significant at 0.05 level of significance. Source: COHEISION Project Clinic Staff Interviews Data (March-June, 2012).

^b^Motivating factors: Motivating factor 1 = clinic physical work environment; Motivating factor 2 = availability of resources and drugs; Motivating factor 3 = financial and extrinsic incentives; Motivating factor 4 = job prospects and career development.

Overall, quality care standards in the surveyed facilities using the NHIA and ESS tools showed a significant positive correlation with most of the staff motivation markers (*P* <0.05) (See Table 4). Thus, as staff motivation levels improve in these motivational factors, efforts towards quality improvement and patient safety will likely improve and vice versa.

## Discussion

Quality care and patient safety in Ghana’s healthcare facilities have been longstanding concerns of health managers, policy makers, patients and civil society [15,16]. Over the years, several interventions have been implemented to improve the situation including motivating health workers through incentives, such as provision of staff accommodation, short promotion intervals, priority for hired purchased cars, paid annual leave and organization of professional development courses [17].

Even though these staff motivation interventions are appropriated towards improving working conditions of health workers, especially in deprived areas, their efficacy towards quality health services delivery has been minimal [18,19].

The baseline results of this study generally showed inadequate efforts towards quality improvement and patient safety in the 64 sampled facilities. According to the the ESS assessment tool, a significant number of the facilities surveyed had ad hoc and emerging uniform risk-reduction activities in place towards quality improvement. Management processes and clinical activities were largely not documented and consistent. Bruce and Killian [15] found similar results in a survey of more than 200 Ghanaian health facilities where only 31% of the facilities had quality assurance teams and documented activities for quality improvement.

Results on the quality care situation are representative of the national NHIS accreditation data where of the 2647 accredited facilities, only 81 (3%) were accredited as grade “A+” and “A” facilities. Up to 4% were granted provisional accreditation because of sub-standard quality care [20].

Perceptions of workplace incentives were generally low including monthly salaries and work allowance. Of the respondents, 55% said they received less than GHC 500 as a monthly salary and more than 80% said they did not receive any form of work allowance outside the regular monthly salary.

Even though some literature suggests that the salary situation for health workers has improved and contributed to decreased annual attrition of skilled health workers in Ghana [21], this study found that satisfaction with the monthly salary remains low among health workers. Since secondary data on actual monthly salaries of the cadre of staff interviewed was not accessed, these self reported salary ranges might have been under reported by respondents as an advocacy for salary increment.

It is also important to mention that the reported low salaries could be attributed to the predominantly low grade cadre of health professionals interviewed in this study. These include nurse-assistants, community health nurses and laborers who are often paid lower salaries and other financial remunerations.

The findings on perceived satisfaction with financial remunerations are critical to understanding and designing financial incentives in the health sector given the important role of financial remuneration in the provision of quality healthcare services.

Financial incentives including the monthly salary are important sources of motivation for workers including health workers. Low salaries of health workers in many African countries, including Ghana, have been cited as a major disincentive for health workers to render good quality care [22] and a push factor for migration [23,24]. Agyepong et al. [4] mentioned low salaries of health workers as a major contributory factor for poor service quality especially in terms of staff attitudes towards patients, informal fee charges and lateness to work. Staff dissatisfaction with working conditions has necessitated engagement in part-time income-generating jobs to supplement regular employment incomes. This practice has been found to have a negative influence on quality service delivery [18].

Even though only 9% of the respondents admitted engaging in part-time work in this study, a significant negative correlation was found between moonlighting and overall efforts towards quality care and patient safety (Coef. = −0.1182, *P* = 0.0369). The reported low engagement in moonlighting in this study could be attributed to under reporting perhaps because the practice is widely perceived to be illegal.

Workload is an important workplace motivating factor especially in developing countries where health sector human and material resources are limited [1]. This study found that, on average, a clinical staff attends to 52 clients a day. The increased financial accessibility for clients following the implementation of the NHIS has been cited as a major contributory factor for increased utilization of orthodox medical services and, therefore, increased the burden on healthcare staff [2,25]. The reported average of 52 clients per staff a day can be attributed to the cadre of surveyed health facilities (clinics and health centres) where existing limited health workers attend to high outpatient department (OPD) and inpatient department (IPD) cases.

According to Bennette and Franco [26], motivation to work is determined by individual, organizational, extrinsic and socio-cultural factors. Over the years, interventions to improve performance of employees have centred predominantly on extrinsic motivators, such as financial rewards. Even though financial incentives are important motivational factors, empirical evidence increasingly shows that without the complement of non-financial incentives, financial incentives alone do not yield the needed performance output from employees [27-30].

This study found that non-financial incentives, such as transportation to work, career development prospects and resource availability at the workplace, are important sources of motivation for staff although perceived by staff to be dissatisfactory. These observations, therefore, necessitate a redesign of more comprehensive staff motivation packages that emphasize these non-financial incentives. These incentives could be prioritized for funding through allocated sums from internally generated funds (IGFs) of health facilities.

The NHIA could also reward best performing health facilities with these staff motivation packages directly or channeled into tariff increment within the concept of performance-based financing (PBF). The assumption is that this would supplement government and individual facility efforts in health worker motivation as a quality improvement strategy. Improving work incentives for health workers will likely stimulate better efforts towards quality improvement and patient safety.

To ensure the operational sustainability of the NHIS in Ghana, currently the concern of many authors [31-33], there is the need to adopt more comprehensive quality improvement interventions that incorporate evidence-based staff motivation strategies. The positive association between staff satisfaction with working conditions and quality care offers stakeholders of health the opportunity to revise existing interventions on staff motivation and quality improvement in Ghana’s primary healthcare system.

Health workers should be treated as internal customers of the health system to enable them to deliver good quality care to patients (external customers). An enhanced level of staff satisfaction with the work environment will likely spill over onto clients and increase satisfaction with service quality.

### Limitations

It is important to acknowledge some limitations associated with this baseline survey. The ESS tool used in this study, though internationally recognized, is not the gold standard for quality assessment and accreditation. The ESS is a risk assessment tool that identifies the current capability of a health facility to slowly or more rapidly move towards higher levels of clinical quality and patient safety. The conclusions are, therefore, identified areas for patient risk reduction and quality improvement.

In addition, the NHIA accreditation data analysed in this study was collected since 2009 and it is possible that the quality situation in these facilities has changed. Moreover, only aggregate NHIS accreditation data were analysed on 64 accredited primary health facilities because the researchers did not have access to the detailed accreditation results. It is therefore possible that the quality situation as presented in this paper is the broader picture on these selected facilities.

Future researchers should include more complex and non-accredited facilities to ascertain differences in staff motivation and efforts towards quality care based on facility size and accreditation status. The current study involved only NHIS accredited primary healthcare facilities.

## Conclusion

Quality care and patient safety standards are generally inadequate in the 64 surveyed primary healthcare facilities. Likewise, staff satisfaction levels with working conditions are low especially in terms of financial incentives and career development prospects. Health workers in private and urban facilities are more motivated by their working conditions than those in public and rural facilities.

It was generally found that staff motivation levels with working conditions positively correlate with quality care and patient safety standards in health facilities, suggesting the need to integrate staff motivation strategies into health facilities quality improvement plans.

### Policy recommendations

Based on the results of this baseline survey, the following intervention areas are proposed for policy consideration:

1. Health managers in public health facilities should invest more in health infrastructure and regular supply of drugs since this was a major source of de-motivation for workers in public facilities. Infrastructural investment should be improved in terms of regular supply of water, provision of modern medical equipment and upgrading of facility OPDs and consulting rooms. These infrastructural investments not only motivate health workers but are also important inputs in delivery of good quality health services needed to attain the health related MDGs.

2. Staff motivation markers as identified in this study should be incorporated into revised NHIA accreditation modules and quality improvement plans for health facilities. The current accreditation tool is predominantly process oriented with minimal criteria on staff efforts towards quality improvement and patient safety. Criteria scores on staff level of motivation with intrinsic and extrinsic motivational factors could form part of the accreditation process for health facilities. Facilities with poorly motivated staff could, therefore, be denied full accreditation.

3. Performance-based remuneration systems should be implemented in private and public health facilities to motivate workers based on quarterly (every three months) performance evaluation outcomes. The current yearly performance appraisals for staff in public health facilities in Ghana are done largely for promotions.

## Endnotes

^a^The NHIA accreditation is done with an accreditation tool that is organized into 12 broad standard areas of which 5 are considered core areas. The five core areas are: (1) range of services; (2) staffing; (3) organization and management; (4) safety and quality management; and (5) care delivery. Depending on the level and category of health facilities, relevant standard areas are applied.

The NHIA accreditation tool employs criteria scores based on facility performance in applicable standard areas. The NHIA criteria score ranges from 0–3, where 3 is scored if all criteria are met; 2 if half or more are met but not all; 1 if less than half are met but not zero; 0 if no criterion is met.

^b^The ESS tool is provided by the SafeCare Initiative, a collaboration of the PharmAccess Foundation, the Council for Health Services Accreditation of Southern Africa (COHSASA), and the Joint Commission International (JCI). The ESS tool is designed to identify the capability of a facility to move slowly or more rapidly towards higher levels of clinical quality and safer patient care according to staff efforts. It consists of 5 primary risk areas and 41 criteria related to quality care and patient safety. The tool has been implemented in African countries, such as Kenya, Tanzania, Ghana and Nigeria.

The five ESS primary risk areas are: (1) leadership process and accountability (7 questions); (2) competent and capable staff (7 questions); (3) safe environment for staff and patients (10 questions); (4) clinical care of patients (10 questions); and (5) improvement of quality and safety (7 questions). Quality assessment scoring is done on 4 levels of effort, from 0 – 3. Higher levels depict better efforts towards quality care and patient safety standards by health facility staff.

Zero is scored when the desired quality improvement activity in a clinic is absent or there is mostly ad hoc activity related to risk reduction. One is scored when the structure of more uniform risk-reduction activity begins to emerge in a clinic. Two is scored when there are processes in place for consistent and effective risk-reduction. Three is scored when there are data to confirm successful risk-reduction strategies and continuous improvement.

## Abbreviations

GAR: Greater accra region; GHS: Ghana health service; GHWO: Ghana health workers observatory; HSHR: Health sector human resource; IPD: Inpatient department; MOH: Ministry of health; NHIA: National health insurance authority; NHIS: National health insurance scheme; OPD: Outpatient department; WHO: World health organization; WR: Western region; MDGs: Millennium development goals.

## Competing interests

The authors declare that they have no competing interests.

## Authors’ contributions

RKA initiated the paper and collected data from the field, cleaned the data, conducted and drafted the initial manuscript. NS reviewed the draft manuscript and contributed to the health facilities quality assessment tool and data collection. PO provided inputs in the methodology part especially on the quality assessment procedure and tools. AO contributed to the statistical analysis and reporting of the analysed results. ENA provided advice on the analysis and interpretation of results, and gave inputs on the policy implications and conclusions made in the manuscript. TRW contributed to the manuscript in terms of the conceptualization of the topic, development of the methodology, discussion of the findings, and the policy recommendations. All authors read and approved the final manuscript.
